# Retinal Vessel Analysis (RVA) in the Context of Subarachnoid Hemorrhage - A Proof of Concept Study

**DOI:** 10.1371/journal.pone.0158781

**Published:** 2016-07-07

**Authors:** Walid Albanna, Catharina Conzen, Miriam Weiss, Hans Clusmann, Matthias Fuest, Marguerite Mueller, Marc Alexander Brockmann, Walthard Vilser, Arno Schmidt-Trucksäss, Anke Hoellig, Marcel Seiz, Claudius Thomé, Konstantin Kotliar, Gerrit Alexander Schubert

**Affiliations:** 1 Department of Neurosurgery, RWTH Aachen University, Aachen, Germany; 2 Department of Ophthalmology, RWTH Aachen University, Aachen, Germany; 3 Department of Diagnostic and Interventional Neuroradiology, RWTH Aachen University, Aachen, Germany; 4 IMEDOS Systems UG, Jena, Germany; 5 Department of Exercise and Health Sciences, University of Basel, Basel, Switzerland; 6 Department of Neurosurgery, Universitätsmedizin Mannheim, University of Heidelberg, Mannheim, Germany; 7 Department of Neurosurgery, Medical University Innsbruck, Innsbruck, Austria; 8 Department of Medical Engineering and Technomathematics, FH Aachen University of Applied Sciences, Aachen, Germany; University of South Florida, UNITED STATES

## Abstract

**Background:**

Timely detection of impending delayed cerebral ischemia after subarachnoid hemorrhage (SAH) is essential to improve outcome, but poses a diagnostic challenge. Retinal vessels as an embryological part of the intracranial vasculature are easily accessible for analysis and may hold the key to a new and non-invasive monitoring technique. This investigation aims to determine the feasibility of standardized retinal vessel analysis (RVA) in the context of SAH.

**Methods:**

In a prospective pilot study, we performed RVA in six patients awake and cooperative with SAH in the acute phase (day 2–14) and eight patients at the time of follow-up (mean 4.6±1.7months after SAH), and included 33 age-matched healthy controls. Data was acquired using a manoeuvrable Dynamic Vessel Analyzer (Imedos Systems UG, Jena) for examination of retinal vessel dimension and neurovascular coupling.

**Results:**

Image quality was satisfactory in the majority of cases (93.3%). In the acute phase after SAH, retinal arteries were significantly dilated when compared to the control group (124.2±4.3MU vs 110.9±11.4MU, p<0.01), a difference that persisted to a lesser extent in the later stage of the disease (122.7±17.2MU, p<0.05). Testing for neurovascular coupling showed a trend towards impaired primary vasodilation and secondary vasoconstriction (p = 0.08, p = 0.09 resp.) initially and partial recovery at the time of follow-up, indicating a relative improvement in a time-dependent fashion.

**Conclusion:**

RVA is technically feasible in patients with SAH and can detect fluctuations in vessel diameter and autoregulation even in less severely affected patients. Preliminary data suggests potential for RVA as a new and non-invasive tool for advanced SAH monitoring, but clinical relevance and prognostic value will have to be determined in a larger cohort.

## Introduction

Patients with aneurysmal subarachnoid hemorrhage (aSAH) face persistently high overall morbidity and mortality. Delayed cerebral ischemia (DCI) defined as clinical worsening or new functional deficit (hemodynamic or metabolic disturbance) has been shown to contribute significantly to cerebral infarction [1], which in turn influences outcome. Management after securing of the offending aneurysm is therefore aimed at early detection of DCI, in order to initiate rescue strategies in due time (induced hypertension, identification of optimum range for autoregulation, angioplasty or intraarterial vasodilation). Detection is facilitated by neurological exam in awake patients. Patients at highest risk for DCI, however, are often unconscious and/or sedated, rendering neuromonitoring considerably more complex and demanding. Each technique employed at this time—invasive or non-/semi-invasive—features its own characteristic set of advantages and disadvantages: digital subtraction angiography (DSA) or CT perfusion (CTP) require transportation of the patient to the imaging unit in order to obtain morphological information (vessel diameter) or hemodynamic information (transit time) at a single point in time. Transcranial doppler (TCD) requires considerably less logistics and can be repeated almost infinitely, but is operator-dependent with moment-to-moment variability and velocity-dependent changes in sensitivity and specificity [2].

In compliance with recent consensus recommendations [3, 4], continuous measurements of perfusion or oxygenation can be performed using CBF-[5, 6] or p_ti_O_2_-probes [7], with cerebral microdialysis adding an estimate of local metabolism [8]. Sophisticated algorithms are able to estimate functionality of cerebral autoregulation, usually by logistic regression analysis of mean arterial pressure (MAP) and intracranial pressure (ICP) or p_ti_O_2_ (PRx, ORx respectively). The significance of autoregulation and individual adaptation of cerebral perfusion pressure has recently been reported [9]. However, calculation usually also requires invasive measuring techniques, limited to a defined, local region of interest.

Ideally, an alternative monitoring technique during the acute stage of SAH (day 2–14) where the risk of neurological or functional deterioration is greatest, would incorporate a repeatable, on-site and non-invasive approach, not only to detect impending hypoperfusion in due time, but also to allow for therapy monitoring such as adaptation of hypertensive treatment or modality and dose of drug-induced vasodilation.

**Fig 1:**
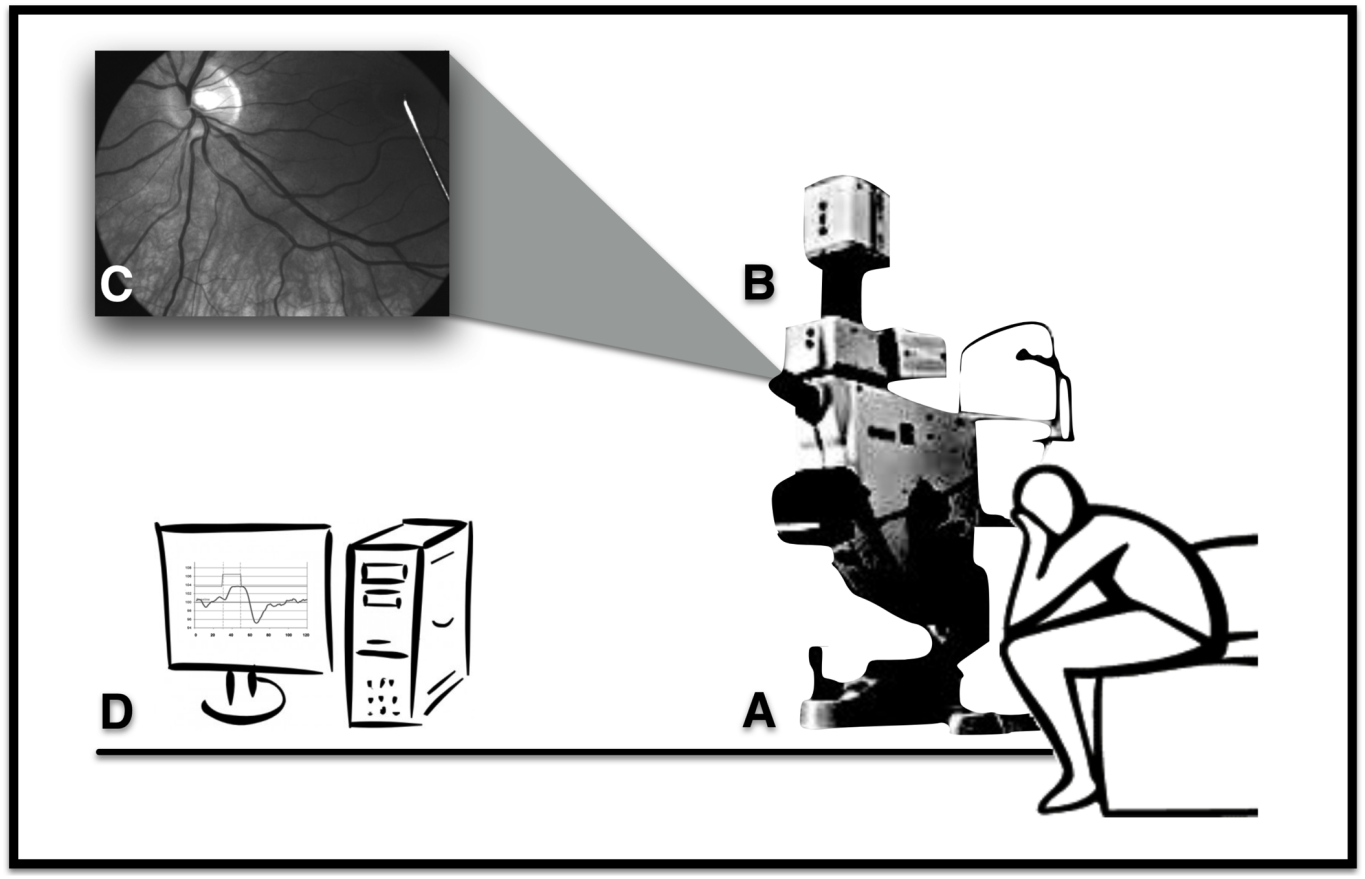
Setup for RVA in SAH patients. The setup for RVA in SAH patients is demonstrated. The patient assumes a sitting position with the head leaned toward the fundus camera (A). An appropriate section of the retina is selected (C). During the exam, a flicker light impulse is generated (B) to test for neurovascular coupling. Vessel diameters are automatically determined in prespecified regions of interest, and a total of three examinations are averaged at a dedicated personal computer (D) which is equipped with the respective analysis software (“RVA” and “VisualIS”, IMEDOS Systems, Jena, Germany; MS Excel, Microsoft, USA).

Retinal vessels are readily accessible for analysis, and vessel diameter is a robust determinant of retinal blood flow. Inspection and photographic documentation of retinal vessel configuration is part of routine ophthalmological practice using commercially available fundus cameras. The “Dynamic Vessel Analyzer” (DVA, Imedos Systems UG, Jena, Germany–use for research only) is a modification of a high end fundus camera (FF450, Carl Zeiss GmbH, Jena, Germany) with tailored hardware and software modules, allowing static and dynamic retinal vessel analysis (RVA, IMEDOS, Jena, Germany). In addition to dimensional analysis, a flicker light stimulation can induce caliber changes of retinal vessels, an index for autoregulatory quality [10]. In principle, healthy vessels dilate with induction of a flicker light impulse as a response to an increase in metabolic demand, so called neurovascular coupling; consequently, flicker dilation (Fig 1) can be employed to describe an important aspect of autoreguation, as published earlier [11, 12] and described in detail [13]. In the past, retinal vessel analysis (RVA) has shown great promise as a predictive screening tool for a variety of systemic disease such as atherosclerosis [14], arterial hypertension [15], myocardial infarction [16] and stroke [17, 18].

So far, there is no data on a potential involvement of retinal vessels in SAH. As an embryological part of the intracranial vasculature, however, retinal vessels may hold a key to a new and non-invasive monitoring technique for intracranial pathologies. Consequently, feasibility and proof of concept of standardized DVA in patients with aneurysmal subarachnoid hemorrhage is the purpose of this study. Time points of examination where chosen within the acute and most critical phase, as well as during follow-up to facilitate a comprehensive characterization of retinal changes during SAH in a time-dependent manner.

## Materials and Methods

This study was approved by the Ethics Committee at RWTH Aachen University, and written informed consent was obtained from all patients (Reference Number EK 069/15).

This prospective pilot study was initiated between September 2015 and January of 2016, recruiting patients both in the acute phase of aSAH and at the time of follow-up. Demographic and clinical data was recorded for all patients, as was the modality of initial treatment and outcome after three months. Intraocular comorbidities or contraindications precluding the application of mydriatic agents—such as a shallow anterior chamber—were excluded before the first examination. For meaningful comparison, an aged-matched cohort of healthy controls was included.

**Fig 2:**
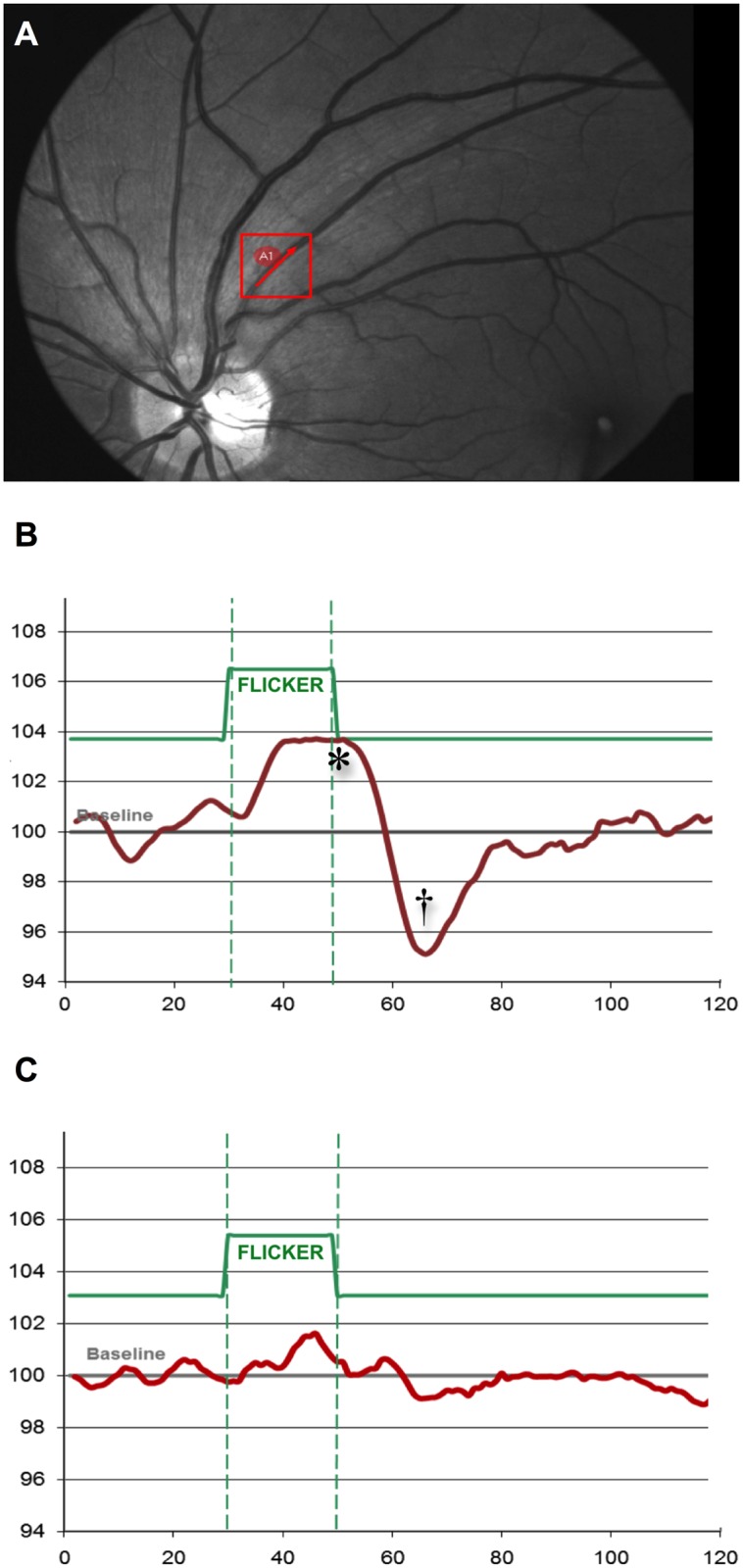
Response curves to flicker light stimulus. A red marking defines the arterial vessel segment under investigation for neurovascular coupling in a photograph of the retinal vascular tree. The example of a healthy control subject (B) shows a characteristic response curve to flicker light stimulus (green) after baseline diameter acquisition. Vessel diameter is depicted as percentage of baseline. With intact autoregulation, a characteristic, primary vasodilation (*) and secondary vasoconstriction (†) is observed with typical latency to the light impulse; an example of a patient with acute, aneurysmal subarachnoid hemorrhage (D) features both a loss of dilation and constriction as well as an overall decrease in amplitude, indicating significant disruption.

**Fig 3:**
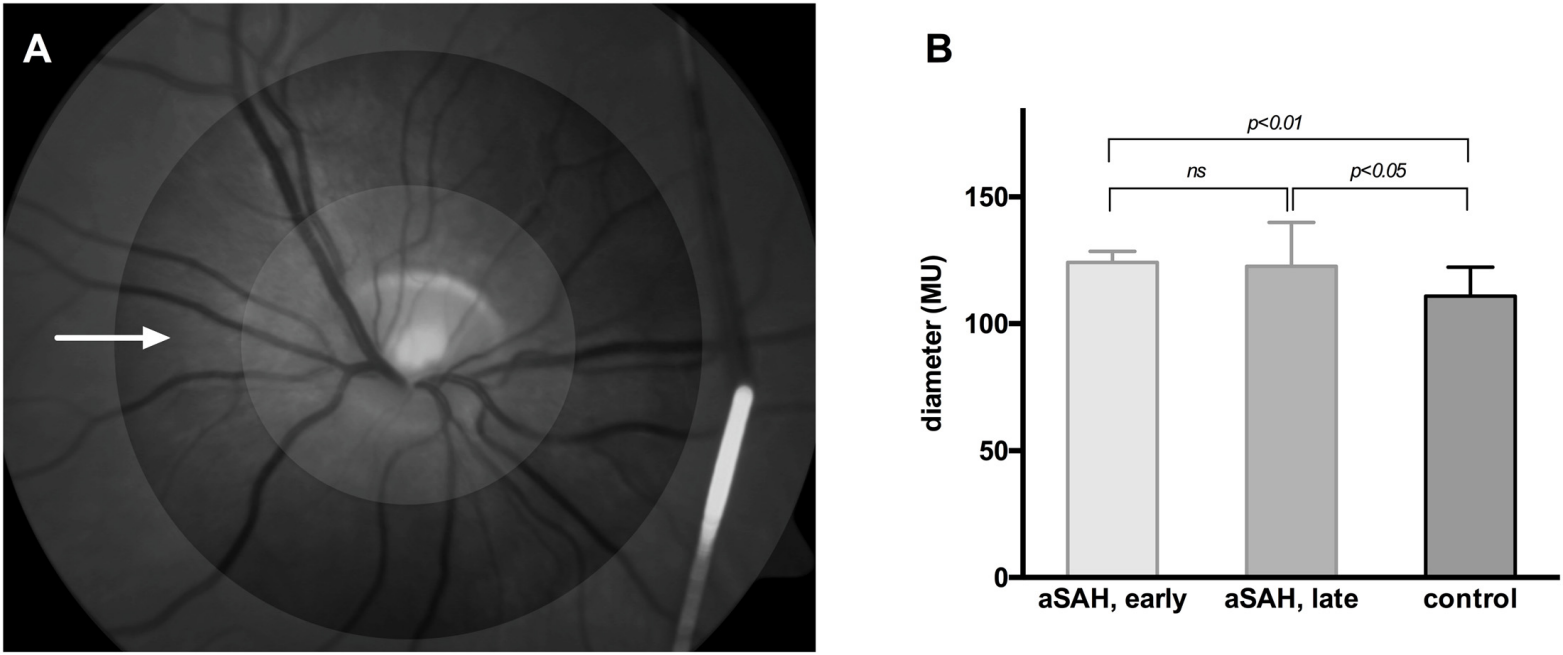
Retinal arterial diameter SAH patients compared to healthy controls. Baseline arterial diameter is determined in a representative vessel segment at a prespecified distance to the papilla (A: white arrow/dark-grey annular segment). For longitudinal investigation in the same patient, the identical position is detected automatically via RVA-software algorithm. In our cohort, arterial diameter is significantly increased in patients with acute SAH when compared to healthy controls (p<0.01); this significance is also present, but to a lesser extent, at the time of follow-up in the late phase after SAH (p<0.05).

Data was acquired using a manoeuvrable DVA for high end fundus imaging (fundus documentation), with dedicated analysis software (RVA, “VisualIS”, Imedos Systems UG, Jena, Germany) for dynamic and dimensional vessel analysis. The individual patient is examined in a sitting position closely facing the fundus camera and instructed to focus on a specific target point after unilateral application of a mydriatic agent. Retinal vessels are brought into view and focus. Flashed retinal fundus pictures are taken for static vessel analysis, and all data is automatically digitally archived for post-processing. For testing of neurovascular coupling and after baseline recording, three sets of flicker light periods (rectangular flash light impulses at 530–600nm at a frequency of at 12.5Hz for a minimum of 20secs each, alternating with 80secs of steady illumination) are completed while the patient is instructed to maintain focus on the target point. For depiction of autoregulatory response, the vessel diameter is recorded continuously in an operator-selected region of interest (Fig 2A) and ultimately plotted as percentage of baseline diameter over time. Implemented algorithms autocorrect for movement, reflection and brightness during video recording. All three sets of flicker light response are averaged in a single response curve to improve signal-to-noise ratio. In healthy subjects with intact autoregulation, a characteristic retinal arterial response curve features primary vasodilation with initiation of the flicker light impulse (Fig 2B, *), reaching a maximum with characteristic latency. Termination of the stimulus is typically followed by a reflectory, secondary vasoconstriction (Fig 2B, †). Shape and amplitude of the response curve are interpreted as an index for quality of vessel autoregulation, and findings are reproducible in healthy subjects [10]. For a quantitative approach to the assessment of neurovascular reactivity, we determined the percentage of maximum dilation and constriction in each patient. For this analysis, raw data of RVA-assessment (absolute vessel diameter changes over time with a resolution of 1 measurement/sec) were extracted and evaluated using a template in MS Excel described previously elsewhere [19]. For illustrative purposes, we also utilized a rudimentary, qualitative classification system, where vessel response (primary vasodilation and secondary vasoconstriction) was judged to be either intact (Fig 2B: healthy control), diminished (reduction of amplitudes) or absent (Fig 2C: loss of dilatory and constrictory component with amplitudal undulation around baseline). For vessel diameter analysis, a prespecified arterial vessel segment was chosen within a representative area of interest equidistant to the papilla (distance to papillary edge between one and two papillary diameters) (Fig 3A). Vessel dimensions are calculated automatically in standardized units (MU), which in an ideal Gullstrand schematic eye correspond to 1μm of absolute diameter. For reliable longitudinal comparison of vessel diameter of an individual patient, the identical vessel segments are automatically detected via a software algorithm at each examination and are visually cross-checked for plausibility before calculation.

## Statistics

Quantitative data is presented as mean ± standard deviation (SD) and as percentage. Student t-test (with/out Bonferroni correction) and Mann-Whitney-test were used for comparison of quantitative parameters (Numbers^®^, Apple Inc., Cupertino, USA; GraphPad Prism^®^, GraphPad Software, Inc, La Jolla, USA). Statistical significance was set at p<0.05; statistical results with p<0.001 were considered highly significant, statistical results with p<0.1 were accepted as a trend.

## Results

During the initial phase of our study, lack of compliance and limited manoeuvrability of the imaging setup prohibited examination of uncooperative, sedated or supine patients. For proof of principle, imaging was performed in awake and cooperative patients in a sitting-upright position. In this selected cohort (n = 15), flicker light examination as the technically most demanding investigation was performed in all but one patient at least once successfully with satisfactory image quality, yielding a total of 14 exams for analysis inclusion (93.3%). Of these 14 exams, six were performed in the acute phase of aneurysmal SAH (aSAH, early: day 2–14) and eight in the late phase after SAH (aSAH, late: mean 4.6±1.7months after SAH). A group of 33 healthy, age-matched controls was included for comparison. Demographic and clinical data as well as outcome is provided in Table 1. One patient presented with a unilateral cataract, but prohibiting examination only in the respective eye. No suspected case of closed-angle glaucoma was identified. No ophthalmological or other complications in association with RVA were recorded during the study period.

**Table 1:** Demography and outcome data of all patients.

Parameter	aSAH, early	aSAH, late	control
	Patients, n (%) or Mean ± SD	Patients, n (%) or Mean ± SD	Patients, n (%) or Mean ± SD
Total	6	8	33
Age (yrs)	56.3±15.9	47.1±11.6	51.3±9.5
Clinical grade			
HH 1–3	6 (100%)	6 (75%)	
HH 4–5	0 (0%)	2 (25%)	
Aneurysm location			
Anterior circulation	5 (83.3%)	7 (87.5%)	
Posterior circulation	1 (16.7%)	1 (12.5%)	
Treatment modality			
Clipping	2 (33.3%)	3 (37.5%)	
Coiling	4 (67.7%)	5 (62.5%)	
Clinical course			
DCI	2 (33.3%)	1 (12.5%)	
Cerebral infarction	1 (16.7%)	1 (12.5%)	
Outcome at time of d/c			
GOS 1–3 (mRS 4–6)	3 (50%)	2 (25%)	
GOS 4–5 (mRS 0–3)	3 (50%)	6 (75%)	

Demographic data is provided for aSAH patients (both early and late), and 33 age-matched controls. Clinical grade, aneurysm location and treatment variables are listed. Clinical course (DCI = delayed cerebral ischemia = clinical worsening in the absence of other underlying pathology; cerebral infarction = new infarction unrelated to clipping/coiling) and outcome (GOS = Glasgow Outcome Score) are also shown. One patient was investigated both in the acute phase, and at time of follow-up. aSAH, aneurysmal subarachnoid hemorrhage; aSAH, early, day 2–14; aSAH, late, mean 4.6 ± 1.7 months after SAH; d/c, discharge; HH, Hunt and Hess grade; GOS, Glasgow Outcome Score; mRS, Modified Rankin Scale.

**Fig 4:**
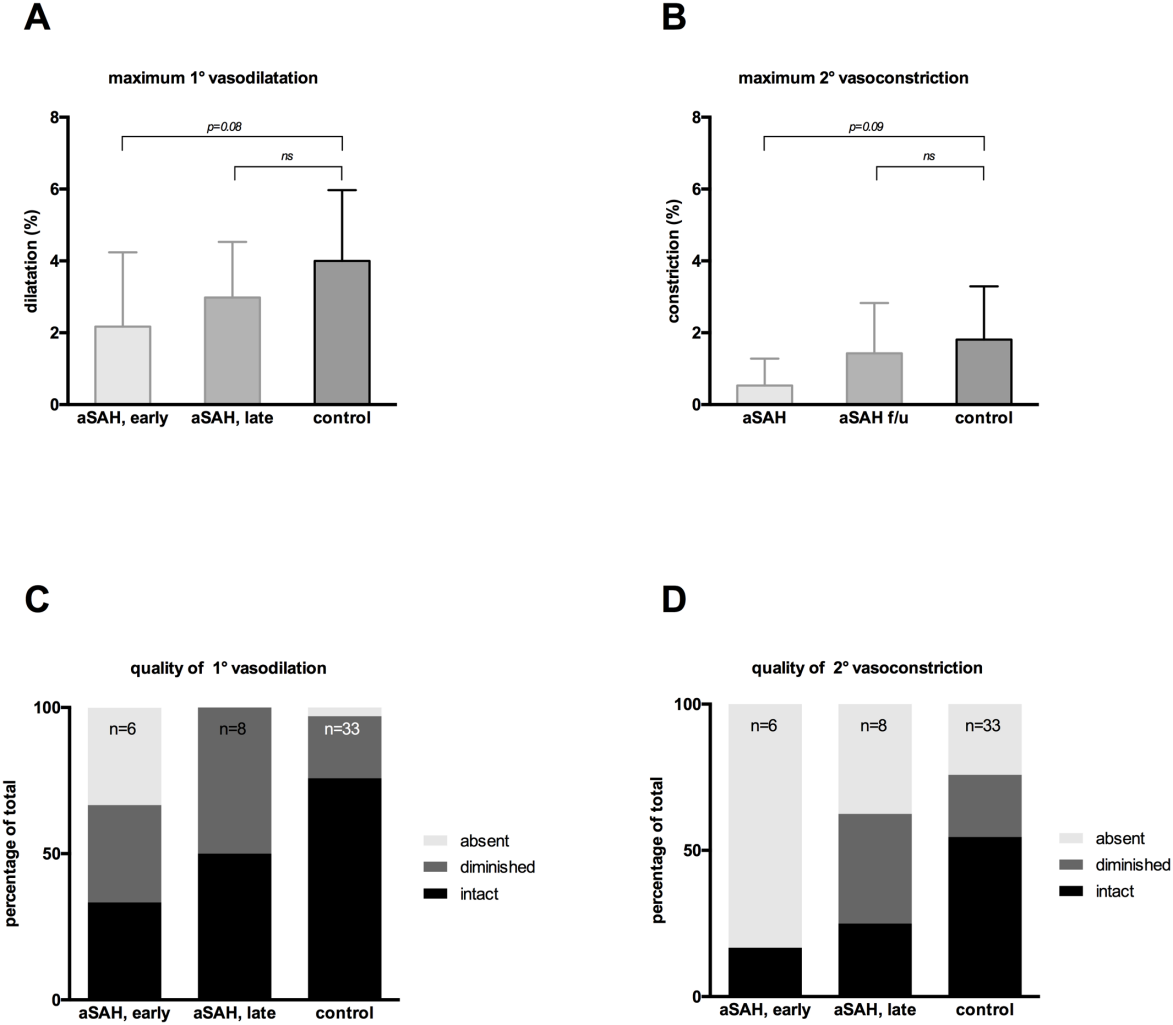
Retinal vessel response to flicker-light for all groups. The response to flicker-light is depicted for all groups. A trend towards a less pronounced primary vasodilation (A) and secondary vasoconstriction (B) is observed, but no significant difference is found late after SAH. Quality assessment of vasodilation (C) and vasoconstriction (D) as judged by amplitude and shape (absent/diminished/intact) illustrates an initial disturbance in the acute phase compared to the healthy control group, with gradual improvement over time (aSAH, late).

Arterial vessels segments were significantly dilated during the acute phase when compared to the control group (Fig 3B: 124.2±4.3MU vs 110.9±11.4MU, p<0.001) with 83.3% of patients being on a prophylactic nimodipine therapy (Table 1). The significance in change of arterial diameter was also present, but less pronounced later after SAH (Fig 3B: 122.7±17.2MU, p<0.05). Following flicker light impulse, a trend towards impaired primary vasodilation and secondary vasoconstriction was observed in the acute phase after SAH when compared to the control group (Fig 4A and 4B: p = 0.08, p = 0.09 resp.). This trend was lost several months after SAH. Quality of primary vasodilation (Fig 4C) and secondary vasoconstriction (Fig 4D) was found to be relatively impaired initially, with the majority of patients featuring a diminished or absent vessel response. Autoregulation showed (partial) recovery over time, as absence of vessel response was observed less frequently in the follow-up examination.

## Discussion

Timely detection of cerebral vasospasm and DCI is essential in order to improve outcome, but can pose a diagnostic challenge, particularly in sedated patients prohibiting neurological examination. For this patient subgroup, advanced monitoring techniques are available, but for the most part are either non-invasive and momentary, or invasive and continuous. Ideally, these modalities could be supplemented with an alternative, non-invasive and repeatable monitoring approach. Changes in vessel caliber and functionality of autoregulation and neurovascular coupling have been shown to be predictive of outcome, and continuous or repeatable assessment of these parameters could potentially be used to further optimize established treatment efforts and eventually improve outcome.

The retinal vasculature shares an embryological origin with strictly intracranial vessels and may in theory lend itself as a new tool for cerebrovascular monitoring. Recent advances combining high-resolution fundus cameras—with electronic recording capability and software support for region of interest selection among others—have facilitated more sophisticated analysis approaches. This study aims to determine the feasibility of retinal vessel analysis (RVA) as a new and non-invasive monitoring technique in patients with subarachnoid hemorrhage, as it is uniquely suited to assess both vessel diameter and functionality of neurovascular coupling.

In awake and cooperative patients, we found that non-invasive measurements with RVA were feasible and provided satisfactory image quality in the majority of cases. Our data suggests that in patients with aneurysmal SAH, RVA can reliably document both vessel dimensions, as well as an estimate of autoregulatory capacity.

**Fig 5:**
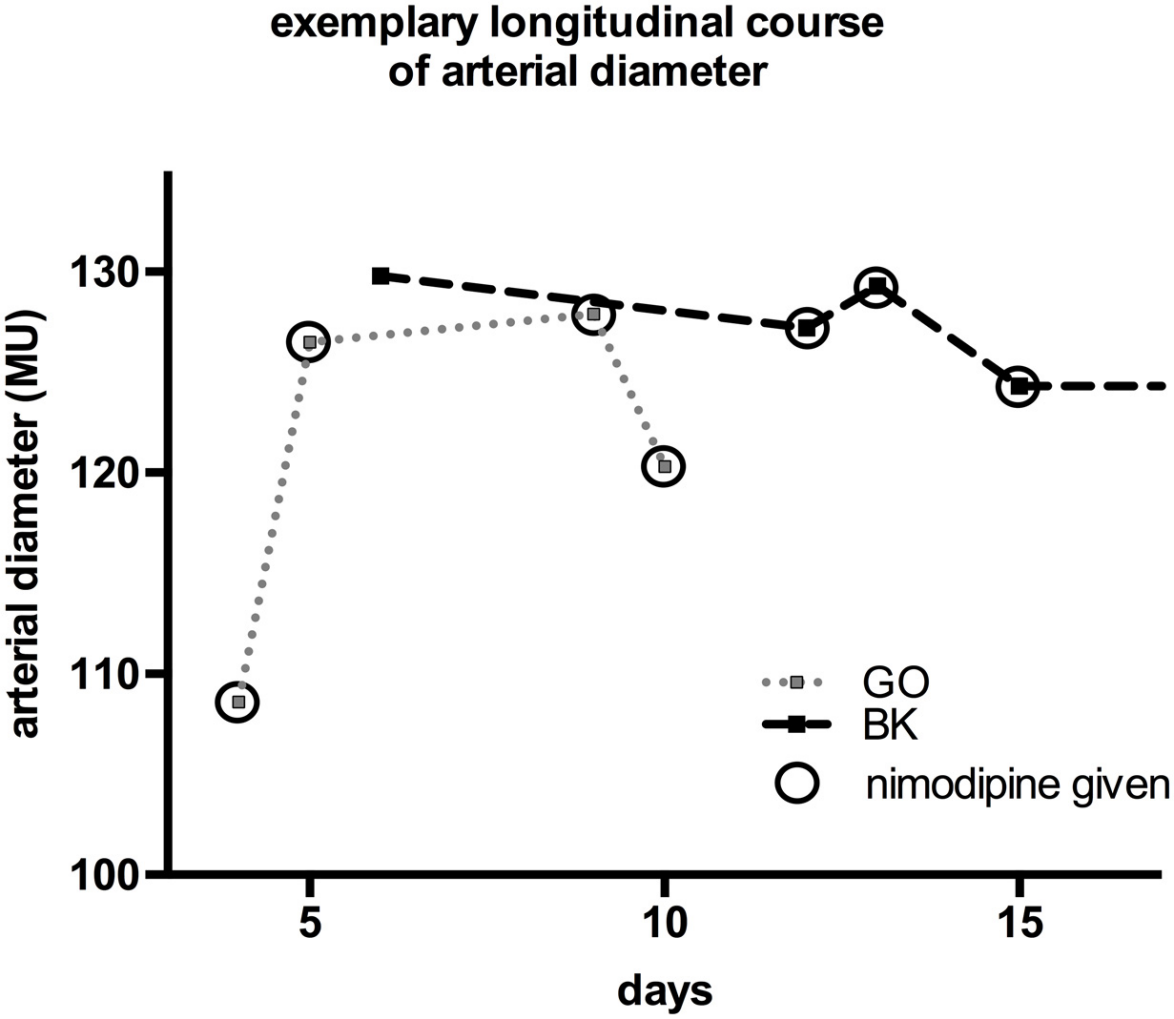
Arterial diameter of two exemplary patients in relation to nimodipine therapy. The longitudinal development of arterial diameter of two exemplary patients (GO, BK) is depicted over time. Increase, decrease and preservation of arterial diameter are observed at initiation or continuation of oral nimodipine therapy.

During dynamic retinal vessel analysis, we observed significant dilation of retinal arteries in the acute phase when compared to a healthy control group. Interestingly, a comparable reflectory response, namely vasodilation, is frequently observed in the early stages of acute or chronic, but still compensated hemodynamic compromise. CBF is maintained through an initial compensatory vasodilation of resistance arterioles, leading to a relative increase of cerebral blood volume [20]. Sufficient compensation at the time of imaging may also be in line with the good clinical condition of patients eligible for RVA in our cohort. Partial restitution of vessel diameter over time was observed, suggesting a demand-driven alteration of arterial diameter in a time-dependent fashion. However, the majority of patients (n = 5, 83.3%) investigated acutely received prophylactic oral nimodipine, which can also influence vessel diameter. The size of our data sets does not allow a more detailed interpretation of the effect of nimodipine. However, two exemplary patients were available for repeated examination during the acute phase; here, longitudinal development of arterial diameter shows both an increase and a decrease in diameter under nimodipine in one (Fig 5: GO), as well as a stagnation of diameter with initiation and discontinuation of nimodipine in another (Fig 5: BK), precluding attribution of a uniform effect on vessel diameter. Furthermore, dilatation of retinal arteries was still observed later in the course of the disease, when nimodipine treatment had been discontinued for several weeks. Acquisition of larger data sets will hopefully shed further light onto the exact role of nimodipine on retinal vessel dimension.

The second aspect under scrutiny using RVA was assessment of autoregulatory function after SAH, both in the acute phase and at the time of follow-up, compared to a control group. Autoregulatory disturbance is frequently observed in the context of subarachnoid hemorrhage using invasive monitoring techniques (as the calculated index of PRx and ORx) and was recently also associated with occurrence of DCI as a contributor for worsening in outcome [9, 21, 22]. In addition, loss of CO_2_ reactivity [23] and impairment of neurovascular coupling [24–26] are characteristical findings in the acute and sub-acute stage after SAH, respectively, providing further evidence for early disturbance of autoregulation after SAH. During RVA testing of neurovascular coupling, two components of a characteristic response curve to flicker light impulse are generally identified: primary vasodilation and secondary vasoconstriction to a flicker-light impulse. Presence and amplitude of a typical, biphasic response are established indicators for autoregulatory quality [27]. In our cohort, we observed an overall rarefication of vessel response, as well as a reduction of response curve amplitude, indicating both a qualitative and quantitative disturbance of (retinal) neurovascular coupling. This impairment was more pronounced acutely, but still present at the time of follow-up. We believe that the presence of this autoregulatory disturbance and its partial recovery over time support the hypothesis of a retinal involvement after SAH. Future investigations will have to correlate previously established means of autoregulation measurement with this alternative approach, in order to verify the relevance of our observation. A longer follow-up with DVA will also determine to what extent retinal changes may persist over time and whether they may have a predictive value for the recovery of frequently observed impairments. Neuropsychological deficits and diffuse visual disturbances after SAH in particular are known to persist over time and only experience gradual recovery if at all. In line with observations in the context of ischemic stroke, we are hopeful that DVA analysis may ultimately facilitate a better prognosis of both outcome and recovery.

## Limitations

Only a limited number of patients could be included in a comparatively short period of time, and only one patient could be examined both acutely as well as at a later follow-up, prohibiting a more detailed analysis and correlation analysis.

Though our findings are reproducible and suggest a characteristic, time-dependent profile, the relevance of our findings and their potential correlation with disease severity, risk profile and overall clinical course remain unclear and have to be the focus of further investigation. Longitudinal assessment of vessel diameter over time, as well as quantification of retinal oxygen saturation and perfusion, and a more detailed analysis of autoregulatory capacity in correlation with other functional parameters (CT perfusion, TCD, p_ti_O_2_-measurements and microdialysis) and clinical course and outcome will have to be included in subsequent investigations.

Also, our cohort of awake and cooperative patients is characterized by a selection bias towards less severely affected SAH patients; this is also illustrated by the very low incidence of DCI and cerebral infarction in our cohort, despite a significant proportion of patients initially presenting with higher Hunt and Hess grade and higher modified Fisher grade. Unconscious patients of higher Hunt-Hess grade, however, are less amenable to clinical exam and generally thought to be at a higher risk for DCI and cerebral infarction, therefore most likely to benefit from additional monitoring; unfortunately and due to practical limitations, these patients could not be investigated during the pilot phase of our study, certainly restricting the generalizability of our results. Regardless of this limitation, however, we were able to observe significant and reproducible alterations, even in our selected group of only mildly affected patients. Current modifications to the camera will improve its manoeuvrability in order to facilitate examination of unconscious and/or sedated patients in supine position. We are hopeful that successful inclusion of more severely affected patients will confirm our preliminary results and provide more information on the expected changes after SAH.

## Conclusions

Retinal vessel analysis is feasible in patients with aneurysmal SAH and reliably detects fluctuations in vessel diameter and quality of autoregulation. Preliminary data suggests potential for RVA as a new and non-invasive tool for advanced SAH monitoring, but clinical relevance has yet to be determined. More data will be collected to provide a meaningful correlation with clinical course and an estimate of its prognostic value.
